# Aging alters interspecific competition between two sympatric insect–parasitic nematode species

**DOI:** 10.1002/ece3.2125

**Published:** 2016-05-05

**Authors:** Farrah Bashey, Tara Sarin, Curtis M. Lively

**Affiliations:** ^1^ Department of Biology Indiana University Bloomington Indiana 47405

**Keywords:** Infectivity, longevity, parasite virulence, species coexistence, *Steinernema*, transmission stage, within‐host competition

## Abstract

1. Interspecific competition can vary depending on the stage, age, or physiological state of the competitors. Competitive ability often increases with age or size; alternatively, senescence can lead to a loss of viability and reduced competitive success. Differences between species in their age‐specific competitive abilities can promote coexistence in the face of substantial niche overlap.
2. We examined two sympatric species of nematodes (genus *Steinernema*) to determine whether their competitive relationship changes as a function of age. These obligately killing insect parasites are known for their broad host ranges and are transmitted from insect to insect via a juvenile stage propagule that is free‐living in the soil. Here, we tested whether the two species differed in the effects of age by examining the mortality of insect hosts infected with young or old transmission stage nematodes of each species. We also performed mixed infections, where an equal ratio of both species was simultaneously exposed to a host, to determine the effect of age on competitiveness.
3. One species showed reduced performance with age, as older propagules were slower at inducing host mortality. In contrast, the other species increased in killing speed with age. In competition, insect mortality rate was predictive of competitive outcome, such that if one species induced considerably faster host death in a single‐species infection, it was competitively dominant in the coinfection. Accordingly, we found a shift in the competitive relationship between the two species with age.
4. Our work demonstrates that species differences in the effects of aging can lead to dramatic shifts in reproductive success. As these effects are realized solely in a competitive environment, both spatial patchiness and temporal niche partitioning may be important for promoting coexistence.

## Introduction

Physiological factors associated with age can play a role in the intensity or outcome of competition. Often juveniles or young adults are more affected by competition than older, larger, and socially dominant adults (e.g., Smith 1981; Eccard and Ylonen 2003). Alternatively, competitive abilities may decline as individuals age and begin to senescence. For example, individuals may no longer be socially dominant or may forage less effectively as they age (Machida et al. 1981; Rockwell et al. 1993).

Senescence effects may also occur in earlier life stages. Many organisms produce dormant propagules, such as seeds or spores, which are known for their long persistence times during unfavorable conditions (Evans and Dennehy 2005). Nevertheless, even these propagules may deteriorate with age. For example, when resting eggs of *Daphnia mendotae* were exposed to conditions appropriate for hatching, older eggs took longer to hatch than younger eggs, and late hatching eggs were more likely to die before maturity (Fox 2007). Moreover, the competitive ability of older propagules may be diminished, as evidenced by the lower end‐of‐season biomass of plants resulting from older seeds of the annual grass *Bromus tectorum*, when grown with an interspecific competitor (Rice and Dyer 2001).

These age‐specific effects on competition may serve to promote species coexistence. If species interactions are patchy in space and time, then shifts in competitive dominance with age could favor different species in different patches. Thus, in the absence of niche differentiation, heterogeneity in the competitive environment driven by the competitors themselves could promote coexistence through a storage effect, if this heterogeneity allows for the persistence of otherwise inferior competitors (Chesson 2000; Amarasekare 2003). Age‐specific effects on competition could also be reflective of different dispersal or resource‐use strategies, which may form the functional basis for coexistence (Angert et al. 2009; Edwards and Stachowicz 2010).

Here, we examine whether aging has an effect on interspecific competition between two species of insect–parasitic nematodes (genus *Steinernema*). Nematodes in this genus have been found to be spatially associated, and they have overlapping patterns of host use (Peters 1996; Spiridonov et al. 2007; Puza and Mrácek 2010). Thus, they provide a good system in which to address how heterogeneity in the competitive environment may facilitate coexistence. Transmission across hosts occurs via a mobile, developmentally dormant, nonfeeding juvenile stage. These juveniles exhibit a wide repertoire of behaviors in seeking out insect hosts and can survive in the soil without a host for several months (Lewis 2002; Strong 2002). Difference between species in how competitive ability changes with age may reflect differing strategies of host use. Our goal is to assess whether age‐specific effects can lead to shifts in competitive dominance.

We focus on multiple isolates of two species collected contemporaneously from the same hillside. Previous work on these nematode isolates has shown that the outcome of within‐host competition between these two species depends on which isolates are paired with each other (Bashey et al. 2013). A major predictor of competitive success is the speed at which each isolate is able to induce insect mortality when singly infecting an insect. Nematode isolates may vary in their killing speed due to genetic differences among the nematodes themselves or their mutualist bacterial symbionts. Additionally, as juvenile nematodes age, they become less successful at colonizing and take longer to kill an insect host (Lewis et al. 1995; Patel and Wright 1997; Patel et al. 1997; Bashey et al. 2007). Does aging in the transmission stage differentially affect these two sympatric nematode species? We first ask whether the insect‐killing speed of each species is affected by age. We then address whether differential aging could promote coexistence by examining whether the competitive outcome between these two species changes as function of the age of the nematodes.

## Materials and Methods

### Nematode life cycle and host use

Nematodes in the genus *Steinernema* form a mutualistic symbiosis with bacteria in the genus *Xenorhabdus*, which help the nematode to kill and digest the insect host, and can be important in determining the outcome of interspecific competition among nematodes (Sicard et al. 2006; Bashey et al. 2013). Upon entering the insect hemocoel, nematodes awaken from their developmental dormancy, begin feeding, and release their symbiotic bacteria from their intestines (Snyder et al. 2007). Insect death results from actions of both the nematode (Goetz et al. 1981; Burman 1982; Simoes 2000) and its bacteria (Herbert and Goodrich‐Blair 2007; Richards and Goodrich‐Blair 2009). Thus, the speed of insect death is due to several properties of the nematode and bacteria that could differ genetically across isolates, as well as being influenced by the physiological state of the parasites and variation in the insect host.

Depletion of energy reserves is a key predictor of changes in infectivity as nematodes age (Lewis et al. 1995; Patel et al. 1997; Patel and Wright 1997). Additionally, the bacterial load carried by each juvenile nematode has been found to decline with nematode age (Lewis et al. 1995; Flores‐Lara et al. 2007). Thus, species differences in initial energy reserves (Therese and Bashey 2012), host foraging behavior (Lewis et al. 1995), or reliance on their bacterial symbiont (Sicard et al. 2006) may shift the relationship between nematode age and speed of insect killing.

*Steinernema* nematodes are capable of infecting a broad range of insect hosts (Peters 1996; de Doucet et al. 1999), and the infective juveniles can persist for months in the soil (Preisser et al. 2005; Torr et al. 2007). Yet, little is known about juvenile survival and host usage in natural populations. Differences in foraging behaviors and reproductive success across different host species create the opportunity for niche partitioning (Lewis et al. 2006; Li et al. 2007; Gruner et al. 2009). However, whether and how this occurs in natural populations still remains elusive as competitive asymmetry can be strong and consistent in direction across several insect species (Puza and Mrácek 2010).

### Nematode isolates

Nematodes were isolated from nine soil cores (8 cm in diameter by 5 cm deep) collected along a 300 m transect at the Indiana University Teaching and Research Preserve at Moores Creek (Monroe County, Indiana) as described in Hawlena et al. (2010). Briefly, larvae of the greater wax moth *Galleria mellonella* were added to soil from each core so that viable *Steinernema* within the soil sample could enter the caterpillars and reproduce. Juvenile nematodes emerging from each caterpillar were considered a separate isolate, and up to three isolates per soil sample were characterized. We identified nematode species by extracting DNA from 1000 infective juvenile nematodes using the spin‐column protocol of the DNEasy Blood & Tissue Kit (Qiagen, USA) and by sequencing the 28S rRNA gene using primers 391, 501–503 and protocols described in Stock et al. (2001). The bacteria associated with each nematode isolate were further isolated as described in Hawlena et al. (2010) and identified to species by sequencing of the 16S rRNA gene using primers 16SP1, 16SP2, SP1, and SP2 and protocols found in Tailliez et al. (2006).

**Figure 1:**
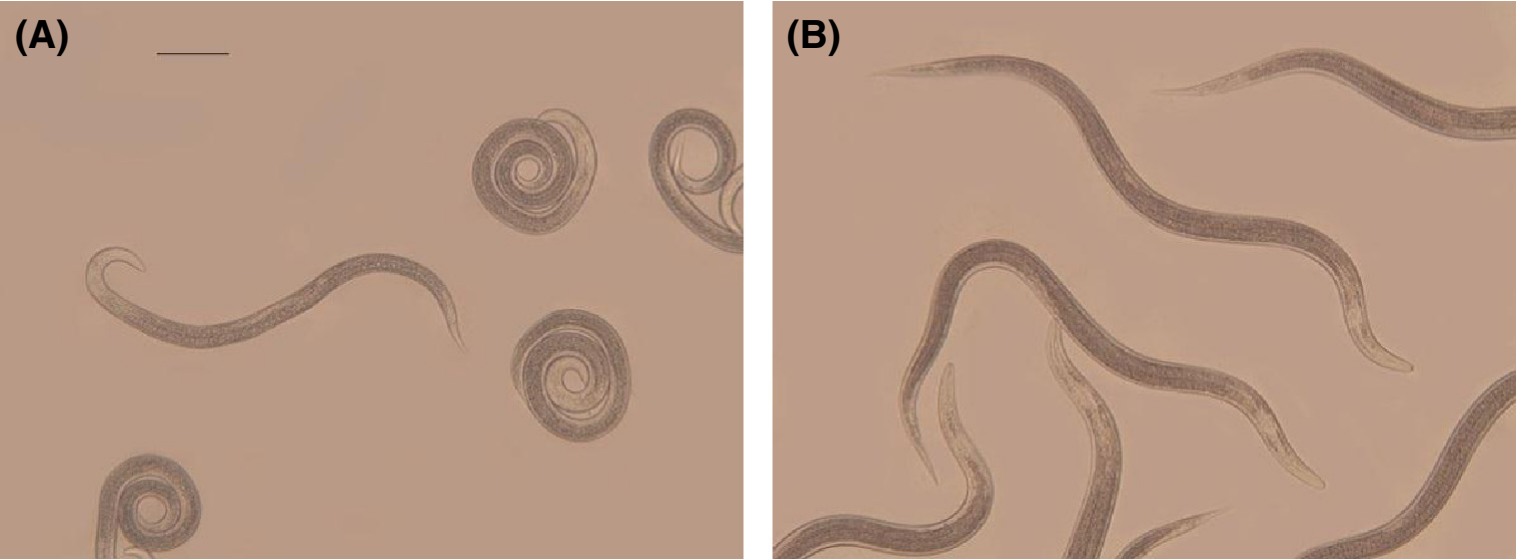
The nematode species used in this study differ in behavior when kept at low temperatures. The Bov nematode (A) rests in tight curls, while the Kop nematode (B) does not. Photo credit L. Schenk.

Seven isolates were found to belong to a *Steinernema* species in Clade III, most closely aligning to *S. kraussei*. These nematode isolates were symbiotically associated with the bacterium *Xenorhabdus bovienii*. For brevity, we will hereafter refer to these symbiotic pairs as “Bov” (Fig. 1A). Nine isolates were found to belong to a *Steinernema* species in Clade IV, most closely aligning to *S. costaricense* (Uribe‐Lorio et al. 2007). These nematode isolates were associated with the bacterium *Xenorhabdus koppenhoeferi* (Hawlena et al. 2010); we will hereafter refer to this symbiotic pair as “Kop” (Fig. 1B).

**Figure 2:**
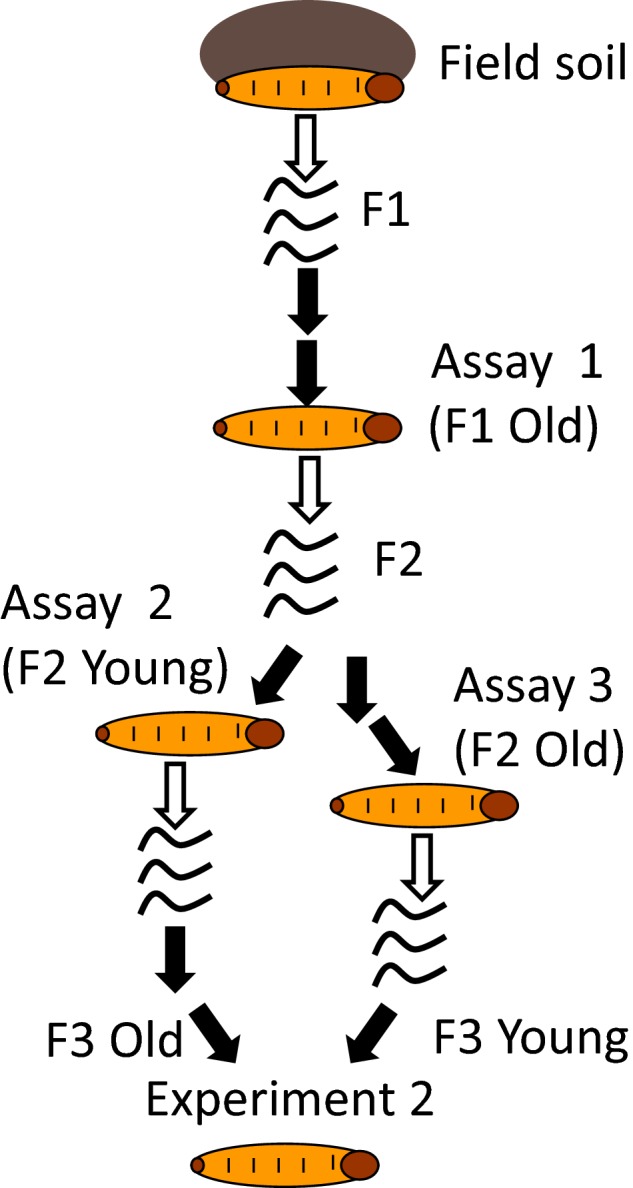
Schematic diagram showing the generation of nematodes used in this study. Arrows represent time, not to scale, within the caterpillar (open) or in storage (closed) after emergence. Experiment 1 consisted of three different assays, performed at different times in the laboratory, in which the nematodes were either old or young when they were exposed to caterpillars and resulted from different host passages (F1 or F2). Experiment 2 compared F3 nematodes of different ages contemporaneously in the laboratory. In both experiments, nematodes of each species were passaged separately. Additionally, Experiment 2 also involved a mixed treatment where nematodes of the two species were simultaneously exposed to the same caterpillar.

These F1 isolates (i.e., the progeny of field collected nematodes) were further propagated in the laboratory by passaging through *G. mellonella* larvae (Fig. 2). F1 nematodes were used to infect caterpillars (as described in Experiment 1), and the emerging nematodes were designated as F2. To maintain outbreeding and to control for environmental differences among caterpillars, subsequent generations of each isolate were propagated by combining the nematodes that emerged from three different caterpillars. F2 nematodes were used to generate F3 nematodes at two points in time so that nematodes of different ages could be studied. Between passages through caterpillars, nematodes were maintained in dH_2_0 and stored in tissue culture flasks at 8°C. Nematodes were observed to be alive and actively moving and were counted just prior to each infection to ensure accuracy of the infective dose.

### Experiment 1: species differences in insect mortality

Previous work on a subset of these nematode isolates had shown that Bov isolates were faster at inducing host death and were competitively dominant to the Kop isolates (Bashey et al. 2011). We set out to determine whether this mortality pattern was (1) consistent across all F1 isolates, (2) repeatable across generations, and (3) affected by nematode age. Accordingly, we used all of the isolates in each of three mortality assays. Assay 1 was conducted with F1 nematodes, which were 11 months old at the time of the assay. Assay 2 was conducted with F2 nematodes, which were 3 months old. Assay 3 was conducted with F2 nematodes, which were 18 months old.

For each assay, each isolate was used to infect 20 caterpillars. Caterpillars were individually placed in a petri dish lined with filter paper, and a drop of 50 nematodes from a single isolate was applied on the dorsum of each caterpillar as described in Bashey et al. (2011). Infected caterpillars were kept at 18°C and examined at intervals for mortality over the following week. After which, caterpillars were transferred to modified White traps (Bashey and Lively 2009) to allow for monitoring and collection of emerging nematodes. Nematodes were allowed to emerge from the caterpillars for approximately 45 days postinfection at which time the caterpillar was discarded and nematodes were transferred to tissue culture flasks.

We used a Cox proportional hazards regression to determine whether species differences occurred in the timing of host death and to examine how this changed across nematode age and generation. The covs(aggregate) option in the PHREG procedure in SAS was used to account for shared variance across caterpillars infected with the same isolate.

### Experiment 2: aging effects on competition

We conducted single and mixed infections using both young and old nematodes simultaneously to test how nematode age affected insect mortality for each species and whether this shifted the outcome of competition. We used the F3 progeny arising from Experiment 1, assays 2 and 3. Thus, nematodes were both passaged twice in the laboratory prior to this experiment, but were of different ages (3 and 18 months) at the time of experiment (Fig. 2). We focused on four isolates in this experiment. Isolates were chosen such that there were no inhibitory interactions between their bacterial symbionts which could influence outcome of competition (Bashey et al. 2013). The first pairing was between Bov isolate 59‐B2 and Kop isolate 86‐K2, while the second pairing was between Bov 44‐B1 and Kop 79‐K1.

For each pairing, there were six infection treatments: Young Bov alone, Young Kop alone, Young Bov Mixed with Young Kop (i.e., Young Mixed), Old Bov alone, Old Kop alone, Old Bov Mixed with Old Kop (i.e., Old Mixed). For each infection treatment, 60 caterpillars were individually exposed (as described for Experiment 1) to a dose of 50 nematodes. These nematodes were either from one isolate alone or from a 50:50 mixture of two isolates. Caterpillars were kept at 18°C and monitored for mortality, emergence and collected as described in Experiment 1.

To determine the nematode species resulting from the mixed treatments, the nematode species was identified by the different behavior exhibited by each species. When kept at 4–8°C, Bov nematodes remain in a tight circle, while Kop nematodes are usually uncurled (Fig. 1). Thus, nematodes from each caterpillar were taken from a refrigerator and photographed immediately to allow for documentation and scoring of the curling behavior. Additionally, bacteria were isolated from a subset of the nematodes (Bashey et al. 2011) to verify the accuracy of the photograph analysis. Nematodes emerging from a single host were classified as either Kop or Bov, as no evidence suggested mixed emergence in this experiment, and prior work has indicated that mixed emergences are rare (Bashey et al. 2011, 2013).

Differences in the time of mortality of each treatment were determined with a Cox proportional hazards regression. Chi‐square tests were carried out to test the association between age and species emergence in both single and mixed infection. Confidence intervals were based on expectations from a random binomial distribution. An ANOVA was carried out to test the relationship between species and the number of nematodes emerging from each host.

## Results

### Experiment 1: species differences in insect mortality

**Figure 3:**
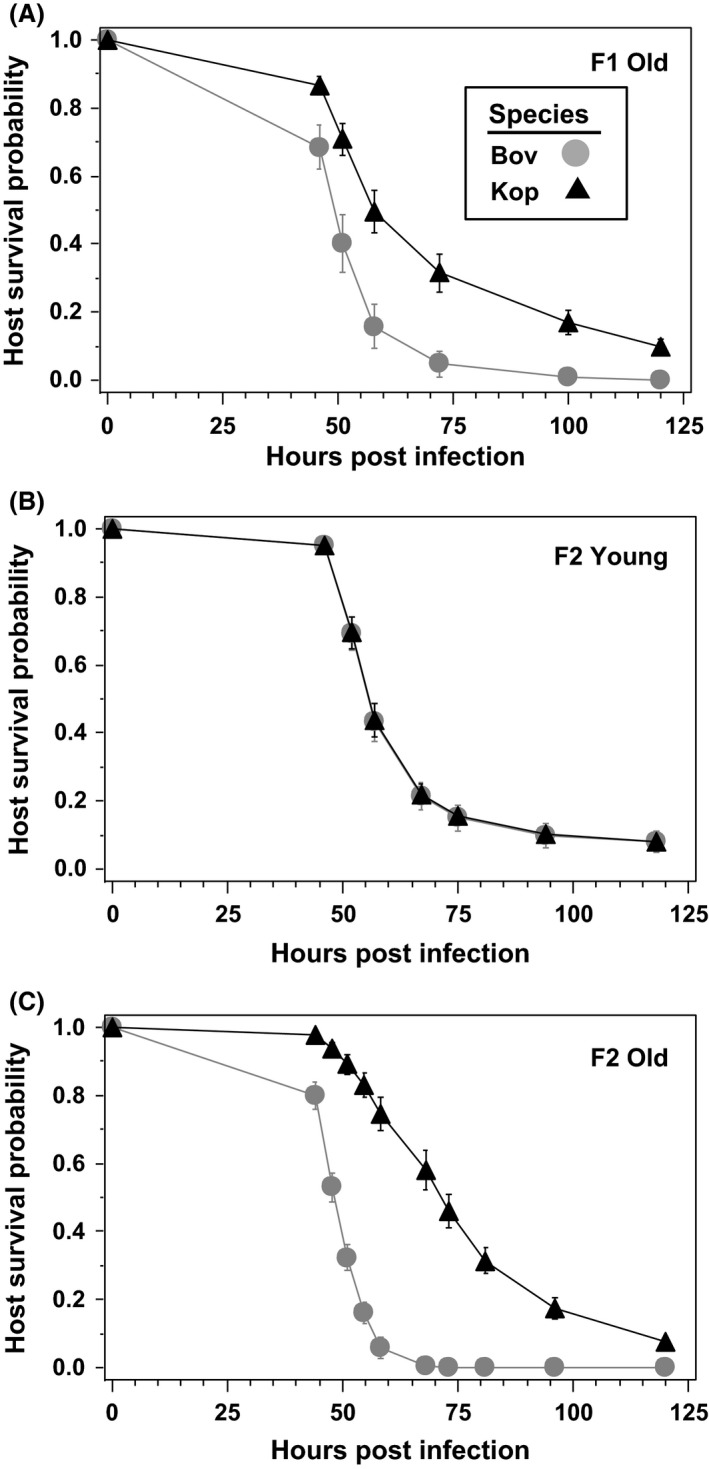
Probability of insect host survival (±1 SE) over time since exposure to Bov (gray circles) and Kop (black squares) nematodes in (A) Assay 1, (B) Assay 2, and (C) Assay 3 of Experiment 1. In each assay, seven different Bov and nine different Kop isolates were each used to inoculate 20 caterpillars for a total of 320 hosts in each assay.

The two nematode species differed with respect to how age affected the rates at which they caused death in the caterpillar host (Fig. 3). In Assay 1, where the nematodes were old (11 months) at the time of inoculation, Bov nematodes were significantly faster at inducing caterpillar death than the Kop nematodes (hazard ratio = 2.63, *χ* ^2^ = 8.10, *P* = 0.0044, Fig. 3A). In contrast in Assay 2, where the nematodes were young (3 months) at the time of inoculation, there were no differences between the species in their speed of inducing host death (hazard ratio = 1.02, *χ* ^2^ = 0.0062, *P* = 0.9370, Fig. 3B). These same F2 stocks of nematodes, however, when used in Assay 3 at 18 months of age, again showed faster killing induced by the Bov nematodes (Hazard Ratio = 9.76, *χ* ^2^ = 66.87, *P* < 0.0001, Fig. 3C). These results suggest that the two species differ in how age affects their ability to successfully colonize a caterpillar. Moreover, analyzing the data across assays indicates that there was no passaging effect on host killing speed between Assay 1 and Assay 3 in either nematode species (Bov: *χ* ^2^ = 1.92, *P* = 0.166; Kop: *χ* ^2^ = 1.56, *P* = 0.211). In contrast, F2 Bov nematodes killed significantly faster when they were old in Assay 3 relative to when they were young in Assay 2 (hazard ratio = 3.79, *χ* ^2^ = 163.54, *P* < 0.0001), while the Kop nematodes killed significantly slower (hazard ratio = 0.41, *χ* ^2^ = 168.02, *P* < 0.0001).

### Experiment 2: aging effects on competition

**Figure 4:**
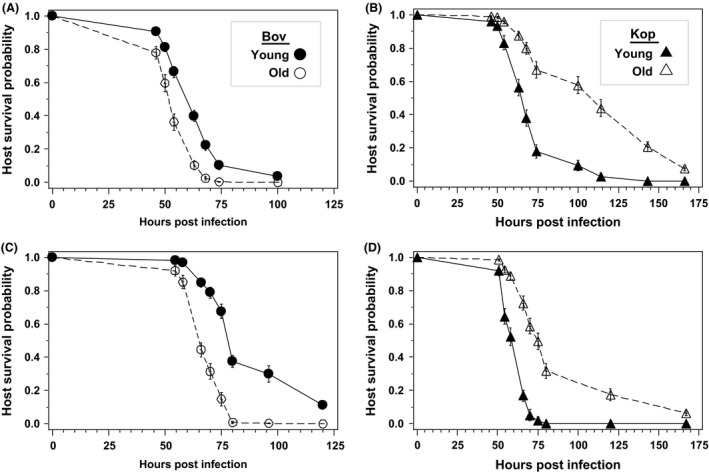
Probability of insect host survival (±1 SE) as a function of hours postinfection for single‐species infections of young (filled symbols and solid lines) and old (open symbols and dashed lines) nematodes performed simultaneously for Bov (circles, panels A and C) or Kop (triangles, panels B and D). Isolates used in pairing 1 are shown on the top two panels (A and B), while isolates used in pairing 2 are shown in the bottom two panels (C and D).

To confirm the age effects on mortality seen in Experiment 1 and to determine whether differential aging could affect fitness, we conducted single and mixed infections using both young (3 months) and old (18 months) nematodes simultaneously. Consistent with the results from Experiment 1, nematode species differed in how age affected the rate at which they induced death in the caterpillar host (Fig. 4). Old Bov nematodes killed hosts more than twice as fast as Young Bov nematodes in pairing 1 (hazard ratio = 2.48, *χ* ^2^ = 25.31, *P* < 0.0001, Fig. 4A) and almost five times as fast in pairing 2 (hazard ratio = 4.86, *χ* ^2^ = 50.80, *P* < 0.0001, Fig. 4C). In contrast, Old Kop nematodes took longer to induce death than Young Kop nematodes (Fig. 4B and D). In both pairings, Old Kop nematodes killed hosts at only 20% of the rate of Young Kop nematodes (pairing 1: hazard ratio = 0.234, *χ* ^2^ = 37.24, *P* < 0.0001; pairing 2: hazard ratio = 0.181, *χ* ^2^ = 43.15, *P* < 0.0001).

Despite their reduced killing speed, Old Kop nematodes were still viable when they infected alone. Host mortality in single infections was >95% for both Kop and Bov nematodes at both ages. Further, Kop nematodes always had greater proportion of successful infections than Bov nematodes (pairing 1: 92% emergence for Kop vs. 66% for Bov, Mantel–Haenszel Statistic = 25.02, df = 1, *P* < 0.0001; pairing 2: 98 vs. 58%, MHS = 56.81, df = 1, *P* < 0.0001). Kop nematodes also had significantly more juveniles emerging from each host, regardless of age (pairing 1: *F*_1,55_ = 77.13, *P* < 0.0001; pairing 2: *F*_1,57_ = 120.92, *P* < 0.0001).

**Figure 5:**
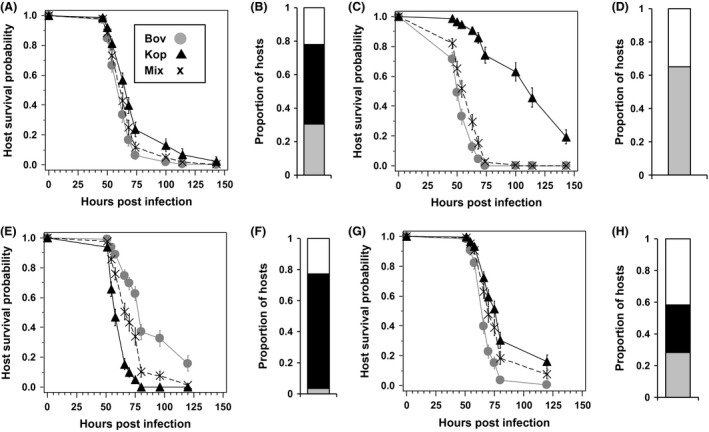
Host survival probability (±1 SE) as a function of hours postinfection for insects infected with either young (A and E) or old (C and G) nematodes of each species alone (Bov, gray circles; Kop, black triangles) or in a 50:50 mixture of the two species (*x*'s). Hosts infected with the mixed‐species treatment resulted in either Bov (gray), Kop (black), or no (white) nematodes emerging as shown for mixtures of young nematodes (B and F) or mixtures of old nematodes (D and H). Pairing 1 is shown on the top panel (A–D), while pairing 2 is shown in the bottom panel (E–H).

Species differed in how age affected their competitive abilities. In pairing 1, Kop nematodes were slightly competitively dominant over Bov nematodes in the Young Mixed infections, emerging from 28 of 46 hosts (Fig. 5B). However, in the Old Mixed infections, Kop nematodes were competitively inferior, emerging from 0/39 hosts (Fig. 5D). This shift in outcome with age was highly significant (*χ* ^2^ = 35.40, df = 1, *P*‐value <0.0001). Pairing 2 showed a similar pattern with Bov nematodes as the emerging species only twice out of 44 hosts in the Young Mixed treatment (Fig. 5F), but then increased to 18 out of 35 hosts in the Old Mixed infection (Fig. 5H). Again, this was a significant association between age of infective dose and species emergence (*χ* ^2^ = 22.66, df = 1, *P*‐value <0.0001).

Species dominance in the mixed treatment was associated with host killing speed. In pairing 1, Old Bov nematodes were 20 times faster at killing their hosts than Old Kop nematodes, and only Bov nematodes emerged (Fig. 5C, Table 1). Similarly, in pairing 2, Young Kop nematodes killed their hosts more than six times faster than Young Bov nematodes, and Kop nematodes emerged from 95% of the hosts (Fig. 5E, Table 1). Across both pairings, as Bov nematodes aged, they increased in host killing speed and in their competitive dominance over Kop nematodes (Table 1).

**Table 1:** Species differences in insect mortality rate and competitive success as a function of age for each pair of isolates

Pairing	Age	Chi‐square^a^	df	*P*‐value	Hazard ratio^b^	Proportion Bov (95% CI)^c^
1	Young	11.94	1	0.0002	1.93	0.39 (0.25–0.55)
	Old	97.34	1	<0.0001	20.50	1.00 (0.91–1.0)
2	Young	80.41	1	<0.0001	0.16	0.05 (0.01–0.15)
	Old	27.11	1	<0.0001	2.82	0.51 (0.34–0.69)

a Results from Cox proportional hazard regressions show significant differences between species in the rate of host death.

b The hazard ratio (Bov/Kop) is comparing the host mortality rate induced by a single‐species infection of nematodes a given age. Values >1 indicate that Bov nematodes killed hosts faster than Kop nematodes when each species was inoculated alone.

c The proportion of hosts with emerging Bov nematodes in the corresponding mixed‐species infection. Confidence intervals are based on the binomial distribution.

## Discussion

Competitive interactions may vary with the stage or physiological state of the individual competitors. These state‐dependent effects can be important in species coexistence if they alter competitive dominance in such a way as to generate times or patches where two species are differentially successful (Chesson 2000; Amarasekare 2003; Kneitel and Chase 2004). Here, we show that the age of transmission stage nematodes has an effect on competitive success. We found that Bov nematodes kill their host faster when old, while the killing rate for Kop nematodes decreases with age (Fig. 4). Furthermore, when one species induced a faster host death when singly infected, it was competitively dominant in the coinfection, meaning that it emerged from more of the coinfected hosts. Thus, Bov nematodes increased in competitive success as they aged, while Kop nematodes decreased in their competitive success (Fig. 5, Table 1).

The shift in competitive ability of the two species with age may reflect their different life‐history or foraging strategies. Kop nematodes produced more, short‐lived propagules, while Bov nematodes produced fewer, long‐lived propagules. In the single infections, Kop nematodes had higher emergence rates than Bov nematodes at both age groups, and they produced more transmission stage nematodes. Kop nematodes also have been found to “jump” and to move greater distances than Bov nematodes (Campbell and Kaya 2002, Bashey et al., unpubl. data). This greater activity of Kop nematodes suggests they may deplete their energy reserves at a faster rate, leading to reduced competitive success at older ages. Thus, Kop nematodes may place a premium on finding more hosts quickly at a cost of reduced longevity.

In contrast, Bov nematodes may be more passive in host finding. As a result, they may maintain competitive ability for longer periods of time. In fact, selection for delayed infectivity in the closely related *Steinernema feltiae* resulted in longer‐lived nematodes that were less likely to immediately infect a host, but were more infective at later ages (Crossan et al. 2007). Additionally, exposure to cold temperatures has been found to initially decrease and then increase infectivity of several species of entomopathogenic nematodes (Fan and Hominick 1991; Griffin 1996). These findings suggest that seasonality may cue infection differently in the Kop and Bov species, such that Bov nematodes become more infectious after over wintering. Thus, the lower host mortality rate caused by Young Bov nematodes could reflect the fact that fewer nematodes are entering the host or successfully resuming development within the hosts than in old Bov nematode infections. Shifts in infectivity with season could reflect optimal shifts in a bet‐hedging strategy for dealing with anticipated changes in stochastic rates of host availability (Fenton and Hudson 2002).

Parasite transmission stages can often persist outside of the hosts for considerable lengths of time. While often the link is made between persistence and greater virulence (e.g., the “Curse of the Pharaoh” hypothesis (Bonhoeffer et al. 1996)), adaptations that enable persistence in the environment may alternatively constrain the parasite's ability to quickly respond to and effectively exploit the host (Caraco and Wang 2008). While differences in response to the host may not influence parasite fitness when singly infecting a host, when in a competitive environment, fitness may be dramatically altered (Fig. 5). So while faster host death increases within‐host competitive success in this system, physiological ties between host‐seeking behavior, longevity in the soil, and recovery within the host may alter the relative growth of each species with time. These links between among‐ and within‐host selection can alter parasite population dynamics and are key to understanding parasite diversity (Bashey 2015).

The importance of dormant stages for promoting species coexistence has been well established both theoretically and empirically (Cáceres 1997; Angert et al. 2009). Our study adds a new dimension by demonstrating that differential senescence in the dormant stage can alter competitive dominance. Given the widespread occurrence of dormancy across all domains of life (Evans and Dennehy 2005; Lennon and Jones 2011), and the potential for aging in the dormant stage to affect future competitive success (Rice and Dyer 2001; Fox 2007), age effects on competitive ability may significantly alter conditions allowing species coexistence in many systems. Moreover, shifts in life‐history strategies, which result in altering senescence in transmission or dormant stages, may provide a common form of temporal niche partitioning.

## Data Accessibility

Data will be made available via DRYAD entry doi: 10.5061/dryad.3c56m

## Conflict of Interest

None declared.
